# Synthesis and Characterization of a Novel Membrane Based on Poly (2-Ethyl Oxazoline) and Poly (Propylene) Graft Copolymer for Potential Food Packaging and Medical Applications

**DOI:** 10.1021/acsomega.5c00605

**Published:** 2025-08-04

**Authors:** Baki Hazer, Zeynep Karahaliloglu, Özgür Keleş

**Affiliations:** † 518002 Kapadokya University, Department of Aircraft Airframe Engine Maintenance, Mustafapaşa Kasabası, Ürgüp, Mustafapaşa, Nevşehir 50420, Turkey; ‡ Zonguldak Bülent Ecevit University, Departments of Chemistry/Nano Technology Engineering, Zonguldak 67100, Turkey; § Department of Biology, Faculty of Science, Aksaray University, Aksaray 68000, Turkey; ∥ Department of Mechanical Engineering and Engineering Science, University of North Carolina at Charlotte, Charlotte, North Carolina 28223, United States

## Abstract

Cost-effective olefin polymers have been producing several hundred million tons of olefins each year. They have material properties suitable for packaging and biomedical applications. Among them, chlorinated polypropylene (PP-Cl) was functionalized with poly­(2-ethyl oxazoline) (PP-polyEtOx) to obtain a biomimetic PP-polyEtOx conjugate polymer material. Poly­(2-ethyl oxazoline) is a water-soluble antibacterial and anticancer polymer. The combination of this bioactive polymer with the elastic properties of polypropylene via graft copolymerization provided a potential active food packaging material. Here, the obtained PP-polyEtOx graft copolymer was characterized structurally using 1H NMR, Fourier transform infrared (FTIR), and X-ray photoelectron spectroscopy (XPS). The water vapor transmission rates of the obtained membranes are better than those of polyethylene terephthalate membrane. Biologic active characterization of the block copolymer was carried out in view of the antibacterial and anticancer properties. The PP-polyEtOx graft copolymers caused a reduction in colony counts for both *S. aureus* and *E. coli* compared to the control. The as-synthesized PP-polyEtOx graft copolymers exhibited an inhibition of viability in HT-29 human colon adenocarcinoma cells.

## Graphical abstract

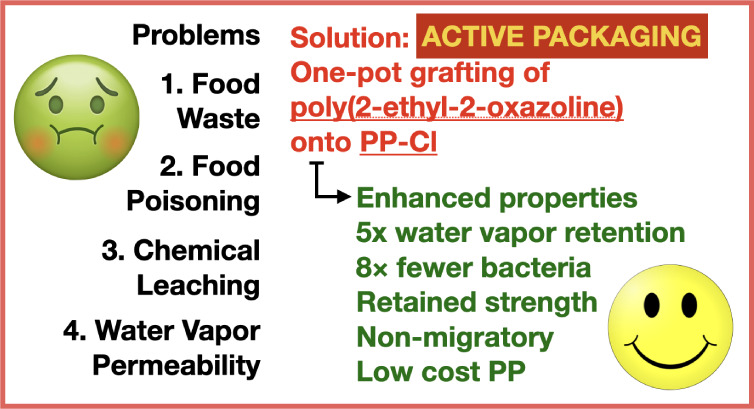

## 1 Introduction

Global food supply chains lose nearly 1.3 billion tons of food each year, while food-borne hazards cause about 600 million illnesses and US $95 billion in productivity losses, underscoring the pivotal role that packaging plays in protecting both resources and public health.^1^ At the same time, food packaging consumes over 40% of all single-use plastics, contributing substantially to the ∼400 million tons of plastic waste generated annually and intensifying calls for climate-neutral, circular-economy solutions.^2^ Extending shelf life by even 10% could divert millions of tons of food from landfills and save billions in disposal and healthcare costs.^3^ Accordingly, next-generation materials must prolong freshness, ensure microbial safety, and minimize environmental impact in tandem. These imperatives have catalyzed a shift from purely passive barrier films toward active and, more recently, intelligent packaging systems designed to interact dynamically with food and its surroundings, setting the stage for the migratory and nonmigratory strategies discussed below.^4–7^

Within this broader sustainability drive, active packaging is rapidly supplanting passive barrier films. These systems interact with the headspace or food surface by lowering microbial load, scavenging oxygen, modulating moisture, and quenching free radicals, thereby extending shelf life and preserving sensory quality.^8–11^ In practice, migratory formats release bioactive agents-such as essential oil components, organic acids, or antioxidants-into the food matrix, while nonmigratory approaches covalently bond or generate these agents in situ on the polymer backbone. Strict migration limits set by U.S. and EU regulations have accelerated the shift toward nonmigratory systems, which reduce leaching, taste/odor taint, and recyclability concerns.^12^ Meanwhile, intelligent packaging-incorporating time–temperature indicators, freshness sensors, or digital trackers-can further minimize waste along complex supply chains by delivering real-time quality feedback. Consequently, the field is actively pursuing biodegradable or chemically up-gradable polymer platforms that can anchor bioactive functions without sacrificing mechanical robustness or processability—a pursuit examined below, and one that ultimately motivates the polypropylene functionalization strategy presented in this work.^13^

Recent research efforts, therefore, focus on truly biodegradable matrices—chiefly aliphatic polyesters such as polylactide (PLA), polyhydroxyalkanoates (PHAs), polybutylene succinate, thermoplastic starch, and polysaccharides such as pectin, and cellulose blends—whose end-of-life degradation can ease landfill pressure.^14–16^ A recent system-dynamics study projects that global production capacity for biobased, biodegradable plastics (a segment dominated by PLA) will climb to ≈1.1 million t/yr by 2030, driven largely by policy-backed demand for compostable packaging.^17^ Yet ester-based films such as PLA readily absorb moisture, and their water vapor permeability can be ∼10^2^-fold higher than that of low-density polyethylene (LDPE)—a disparity that accelerates hydrolytic embrittlement and undermines barrier performance.^18^ State-of-the-art barrier upgrades—such as ultrathin SiO_*x*_ or AlO_*x*_ nanocoatings, clay-based nanolayers, or conventional polymer/aluminum multilayer coextrusions—can certainly suppress oxygen and moisture ingress; however, they add extra processing steps and material costs, complicate mechanical sorting and delamination during recycling, and still provide only a passive barrier that lacks any intrinsic antimicrobial activity.^19^ Consequently, even advanced “bioplastic” wraps still fall short of shelf-life targets for high-moisture foods without auxiliary sachets or secondary barriers. Against this backdrop, upgrading readily available polypropylene with covalently anchored bioactive side chains offers a complementary path-maintaining polyolefin-level performance while introducing the nonmigratory functionality associated with next-generation biodegradables.

Petroleum-based plastics such as polypropylene, polyethylene, and polyvinyl chloride continue to be widely used in food packaging applications due to their excellent mechanical and physical properties. However, their lack of biodegradability and intrinsic biological activity limits their functionality. Therefore, functionalizing these polymers with natural compounds has emerged as an effective strategy for developing active food packaging materials.^20^ Various naturally derived substances have been explored to enhance biological activities through synergistic effects, including tannic acid, caffeic acid, vanillic acid, cinnamic acid, coumaric acid, and naringin.^21,22^ Other noteworthy functionalizing agents reported in the literature are abietic acid, bovine serum albumin, morphine, indole, lysozyme, monoethyl fumarate, aspirin, menthol, and lipoic acid.^23^ Building on these approaches, the following examples were reported.

For instance, biologically active molecules impart antioxidant and antibacterial properties to vinyl polymers.^24^ Also, cost-effective polypropylene films were successfully functionalized by photografting hydroxyethyl methacrylate monomer, which was further esterified with caffeic acid, resulting in antioxidant-active food packaging materials.^25^ Another promising approach involves the use of poly­(2-ethyl oxazoline) (polyEtOx), a water-soluble, biocompatible, and antibacterial polymer synthesized via the cationic polymerization of 2-ethyl oxazoline (EtOx), a nitrogen-containing heterocyclic monomer.^26–31^ When combined with biodegradable polymers, polyEtOx can enhance their biodegradability.^32^ Additionally, in aqueous solutions, polyEtOx exhibits lower critical solution temperature (LCST)-type phase transitions, with reported cloud points varying significantly (e.g., from about 36–80 °C or 62–100 °C), depending on molecular weight, polymer concentration, polymer architecture (homopolymer vs copolymer), and other solution-specific conditions.^28,31^ This thermoresponsive behavior is often compared to poly­(*N*-isopropylacrylamide) (pNIPAM); however, exact transition temperatures and behaviors can differ substantially between these polymer systems.

PolyEtOx is a hydrophilic member of the poly­(2-alkyl-2-oxazoline) (PAOx) family, whose cationic ring-opening polymerization imparts exceptional structural diversity and, consequently, finely tunable thermal, solution, and biological properties.^33^ This versatility has already been leveraged to create high-capacity drug-delivery systems, SARS-CoV-2 vaccine excipients, and peptide-mimetic antifungal platforms, emphasizing the protein-like bioactivity of PAOx materials.^33^ PAOx chains can also mimic host-defense peptides, conferring protease-resistant, broad-spectrum antibacterial action-highly beneficial for food-contact and biomedical membranes.^34^ Meanwhile, PAOx-based hydrogels have proven capable of supporting multicellular spheroids and intestinal organoids without supplemental extracellular-matrix proteins, confirming their cytocompatibility and functional similarity to native protein scaffolds.^35^ The incorporation of reactive handles (e.g., alkenes, alkynes, and azides) further enables straightforward postpolymerization functionalization, allowing chemists to tailor macromolecular architecture for specific applications.^33^ Because PEtOx combines pronounced hydrophilicity with negligible cytotoxicity, it serves as a proteolysis-resistant PEG-substituting modifier for surfaces and matrices. Grafting PEtOx onto mechanically robust yet hydrophobic polypropylene thus provides a direct route to membranes that unite high structural strength with biologically relevant functionality. Importantly, the simple one-pot grafting route described here can be carried out on existing polyolefin processing lines, providing an immediately scalable upgrade rather than a long-term materials overhaul. By uniting polyolefin-level barrier strength with covalently anchored antibacterial and anticancer activity, the resulting PP-*g*-PEtOx films offer a practical, regulation-compliant path toward safer food supply chains and value-added biomedical membranes.

In this study, polyEtOx was grafted onto chlorinated polypropylene to synthesize a polypropylene-polyEtOx graft copolymer. Leveraging the inherent biological activity of polyEtOx, we systematically explored potential applications of this amphiphilic graft copolymer for active food packaging and biomedical contexts, including potential anticancer applications. The copolymer’s mechanical properties, water vapor transmission characteristics, antibacterial efficiency, and anticancer activity were evaluated comprehensively. These combined assessments highlight how the developed graft copolymer effectively integrates barrier performance with beneficial biological functionalities, establishing its suitability as a multifunctional material.

## 2 Experimental Section

### 2.1 Materials

PP-Cl (CAS 68442–33–1) [26% Cl; Mn­(GPC) = 69000 Da, polydispersity (Đ) = 3.43], 2-ethyl oxazoline (CAS 10431–98–8) (EtOx), and tetrahydrofuran (THF) (CAS 109-99-9) were supplied by Sigma-Aldrich and were passed through an Al_2_O_3_ (CAS 1344-28-1) column before use. Methyl p-toluene sulfonate (CAS 80-48-8) (MepTs), acetonitrile (CAS 75-05-8) (AcCN), sodium hydride (NaH, 60 wt % in oil) (CAS 7646-69-7, 8042-47-5), and all other chemicals were purchased from Sigma-Aldrich.

ATCC 25923 of Gram-positive (*S. aureus*) and ATCC 25922 of Gram-negative (*E. coli*) bacterial strains were cultured. For flow cytometry and ROS analysis, the human colorectal adenocarcinoma cell line (HT-29, ATCC HTB-38) was used, and DMEM-F12, fetal bovine serum (FBS), and penicillin–streptomycin were purchased from Biological Industries, Israel. H2DCF-DA (2’,7’-dichlorofluorescein diacetate), used for reactive oxygen species (ROS) analysis, was obtained from Sigma (Sigma-Aldrich, USA).

### 2.2 Synthesis of Poly (2-Ethyl Oxazoline) (PolyEtOx)

Cationic polymerization of 2-ethyl oxazoline was carried out by the modified procedure reported in our recently published article.^36,37^ Briefly, 2-EtOx (5.39 g, 54 mmol) and MepTs (0.39 g, 2.1 mmol) were dissolved in AcCN (1.46 g). Argon was passed through the solution for 1 min. The solution was kept in an oil bath at 85 °C for 20 h. To obtain hydroxyl-functionalized poly­(EtOx), the reaction was terminated by adding 1 mL of a solution of KOH in methanol (2%) and then the polymer was precipitated in 100 mL of diethyl ether. The white solid polymer was dried in a vacuum oven at 40 °C for 24 h. Yield: 4.54 g. Molar mass: Mn 1570 g/mol; dispersity, Đ, 1.26. Additional poly­(EtOx) samples were obtained with Mn between 1300 and 1813 Da and Đ values between 1.04 and 1.29.

### 2.3 Synthesis of PP-*g*-(PolyEtOx) Graft Copolymer Membranes

The hydroxyl end of poly­(EtOx) was reacted with NaH in THF solution to obtain poly­(EtOx) sodium oxide. Then, it was poured into a THF solution of PP-Cl while continuously stirring at room temperature. After 24 h of stirring, the unreacted NaH was neutralized by introducing 5 mL of methanol. The solution was precipitated into 0.3 L of 0.1 M aqueous HCl. The precipitated PP-*g*-PEtOx graft copolymer was washed with distilled water several times and dried under vacuum at 40 °C for 24 h. Table 1 contains the amounts of the reagents and GPC results. The polymer derivatives were precipitated as flocculants. Then, they were dried and redissolved in chloroform (2 g in 20 mL of CHCl3) to prepare a solvent-cast polymer membrane. For this, the chloroform solution of the polymer was poured into a Petri dish (diameter 7 cm). A cardboard was placed over it overnight keeping the solvent to evaporate and leaving the polymer film in the Petri dish. The film was then removed from the glass container.

**Table 1:** Amounts of the Reagents and the GPC Results for the Synthesis of PP-*g*-PEtOx^a^

Code	PP-Cl (g)	PolyEtOx (g) (%)	NaH (g)	Yield (%)	W.Upt. (%)	Mn (kDa)	Mw (kDa)	Đ	PolyEtOx content%
PP-PolyOx-1	1.46	1.04 41	0.27	55	18	112	262	2.33	22
PP-PolyOx-2	1.58	2.12 57	0.53	36	16	95	262	2.76	18
PP-PolyOx-3	2.60	2.08 44	0.24	35	23	101	252	2.50	22
PP-PolyOx-11	2.81	0.83 22	0.22	44	14	93	258	2.76	22
PP-PolyOx-12	1.36	1.08 44	0.29	48	15	80	234	2.94	25
PP-PolyOx-13	2.00	0.55 21	0.21	76	17	100	255	2.54	22
PP-PolyOx-14	1.60	2.44 60	0.62	23	20	76	178	2.34	45
PP-PolyOx-15	1.60	0.73 31	0.43	45	16	72	176	2.45	n.d.
PP-Cl					9	69	237	3.43	-

a Wa. Upt.: water uptake, Đ: poly dispersity, N.D.: not determined.

### 2.4 Characterization

1H NMR spectra of the products in CDCl3 solution were recorded using an Agilent 600 MHz NMR (Agilent, Santa Clara, CA, United States) spectrometer equipped with a 3-mm broadband probe. FT-IR spectra of the polymer samples were recorded using a Bruker Model Tensor II instrument with the ATR technique in the transmissive mode and a scan range of 450 to 4000 cm^–1^. A Viscotek GPCmax auto-sampler system, consisting of a pump, three ViscoGEL GPC columns (G2000H HR, G3000H HR, and G4000H HR), and a Viscotek differential refractive index (RI) detector, was used to determine the molecular weights of the polymer products in THF solution. A calibration curve was generated with five polystyrene (PS) standards of molecular weights 2960, 8450, 50,400, 200,000, and 696 500 Da with low polydispersity. Data were analyzed by using Viscotek OmniSEC Omni 01 software.

To determine the elemental composition, a Thermo Scientific K-Alpha X-ray photoelectron spectroscope (XPS) was used. This instrument uses a 400 nm diameter beam and a monochromatic Al–Kα X-ray source.

### 2.5 Antibacterial Activity of PP-2-ethyl Oxazoline (PP-PolyEtOx) Membranes

Prior to the plaque counting test, the membranes were sterilized under UV radiation for 30 min. The bacteria (*S. aureus* and *E. coli*) were initially cultured in nutrient broth medium under shaking conditions (250 rpm) at 37 °C overnight. After incubation, the density of bacterial strains was adjusted to 0.5 McFarland (10^8^ CFU/ml) (OD600 = 0.08–0.1) with PBS. The sterilized PP-PolyEtOx membranes were then exposed to each bacterial solution at 37 °C for 24 h. The following day, the membranes were taken out of the bacterial suspensions and washed with PBS to eliminate unattached bacteria. To quantify the adhered bacteria, we serially diluted the bacterial suspensions 10-fold, plated them onto nutrient agar, and incubated at 37 °C for 24 h under static conditions. The colonies on the agar plates were then counted to determine the final CFUs.

### 2.6 Flow Cytometric Apoptotic Assay

Flow cytometry analysis was performed using a human colorectal adenocarcinoma cell line (HT-29, ATCC HTB-38), which was cultured in DMEM-F12 supplemented with 10% (v/v) fetal bovine serum and 1% penicillin/streptomycin in a 5% CO_2_ humidified incubator at 37 °C until the cells reached approximately 80% confluency. Meanwhile, the sterilized membranes (1 × 1 cm^2^) were soaked in 5 mL of cell culture medium overnight. Following this treatment, the medium was withdrawn and the reaction was preserved. The next day, the cell medium was replaced with the preserved medium, and the cells were incubated overnight. Subsequently, Annexin V/propidium iodide staining was carried out according to the manufacturer’s instructions. Briefly, the cells were washed twice with PBS and centrifuged after being harvested by trypsinization. The cell pellets were collected in an Eppendorf tube and resuspended in 400 μL of Annexin V binding buffer. A mixture of 5 μL of FITC-Annexin V and 10 μL of propidium iodide was added to the Eppendorf tubes and incubated for 15 min in the dark. The samples were analyzed using a FACSCalibur (BD Biosciences, Heidelberg, Germany).

### 2.7 In Vitro Intracellular ROS Detection

To detect the intracellular ROS level of HT-29 cells treated with PP-PolyEtOx membranes, the ROS-sensitive probe 2“,7”-dichlorodihydrofluorescein diacetate (H2DCFDA) was used and dissolved in DMSO to obtain 10 mM stock solutions. For this analysis, the cells were initially seeded into 6-well plates at a density of 1 × 10^4^ cells per well and incubated at 37 °C in 5% CO_2_ atmosphere until 80–100% confluency (2–3 days). As mentioned in the flow cytometer analysis section, a preserved medium was prepared, and the cell culture medium was replaced with this stored medium after 24 h incubation. Next day, the cells were incubated with 10 mM H2DCFDA for 30 min in the dark and washed twice with PBS. Then, the cells were collected by trypsinization and washed to remove H2DCFDA impurities. The pellets were suspended in PBS at pH 7.4, and finally, the cells were analyzed by using FACSCalibur (BD Biosciences, Heidelberg, Germany).

### 2.8 Mechanical Properties

Onalkon Tensile Testing machine, using a 5 kg load cell and a stretch speed of 20 mm/min, was used for tensile property characterization of the individual THF cast film samples with dimensions of 0.16 × 10 × 50 mm. All samples (*n* ≥ 3) were dried at room temperature under vacuum for 10 days prior to measurement. Standard deviation in mechanical strength and elongation was calculated by the machine program using the following eq 1

1 $$\sigma =\sqrt{\frac{\sum {\left(X-\mu \right)}^{2}}{N}}$$

Where σ is the population standard deviation, *X* is each value, μ is the population mean, and *N* is the number of values in the population.

### 2.9 Water Vapor Transmission Rate

The water vapor transmission rate (WVTR) of the films was measured by using the gravimetric cup test method (ASTM E96, 2024). After the film specimens with a diameter of 60 mm were prepared, they were conditioned in a climate cabinet at 23 °C and 50% humidity for at least 2 days. The thicknesses of the film samples were measured at five different points by using a digital micrometer. Then, the films were placed in the mouth openings of poly­(methyl methacrylate) cups containing dry desiccants and screwed tightly around the edges. Weight changes were monitored at 2-h intervals for 2 days in these WVP cups, which were kept in a climate cabinet at 23 °C and 50% humidity. The water vapor transmission rate was calculated according to eq 2 given below.^38^

2 $$\mathrm{WVTR}=\frac{G}{t\cdot A}$$

where *w* is the weight gained (gram); *t* is the time (second); and *A* is the area of the film exposed to water vapor permeation (m^2^).

## 3 Results and Discussion

Grafting reactions of polyEtOx to PP-Cl were successfully carried out. The chemical reaction is illustrated in Figure 1.

**Figure 1:**
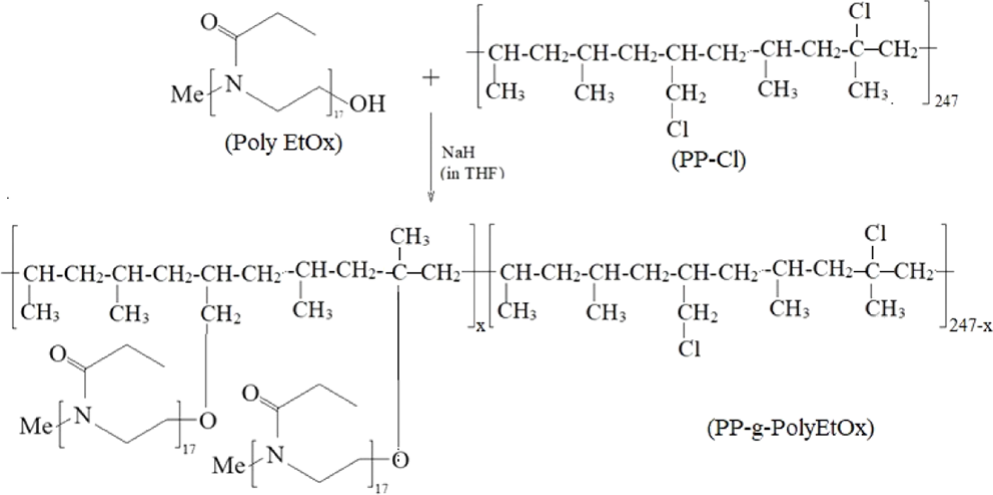
Synthesis of the PP-*g*-PolyEtOx graft copolymer.

PP-*g*-PolyEtOx graft copolymers were well-characterized structurally using 1H NMR, FTIR, and XPS. ^1^H NMR spectra of the as-synthesized PP-*g*-PolyEtOx graft copolymers can be seen in Figure S1. In the PP-Cl spectrum, there were mainly two types of protons: −CH_2_–Cl and −CH–Cl) and aliphatic protons (−CH_2_ and −CH) present in the main chain of PP-Cl. The resonance signals of the aliphatic protons were located between 1.71 and 2.81 ppm, whereas Cl attached groups were located at 3.55 and 3.68 ppm. After graft copolymerization, partially chloride groups were partially exchanged with polyEtOx. The characteristic chemical shifts of the polyEtOx and PP-Cl blocks were mostly overlapped, such as at 3.4–3.7 ppm (C**H**_2_-Cl, -C**H**-Cl for PP-Cl; −N-C**H**_2_-C**H**_2_- for polyEtOx) and 0.7–1.6 ppm, except for the special signal at 2.2–2.5 ppm for polyEtOx (CH_3_–C**H**_2_–C­(O)−). Therefore, the 1H NMR spectra were enlarged between 3.3 and 4.4 ppm. As PP-Cl shows a very narrow peak between 3.50 and 3.48 ppm, PP-poly EtOx graft copolymers exhibited a broadened peak between 3.40 and 3.50 ppm, which was attributed to the existence of polyEtOx segments (Figure 2I).

**Figure 2:**
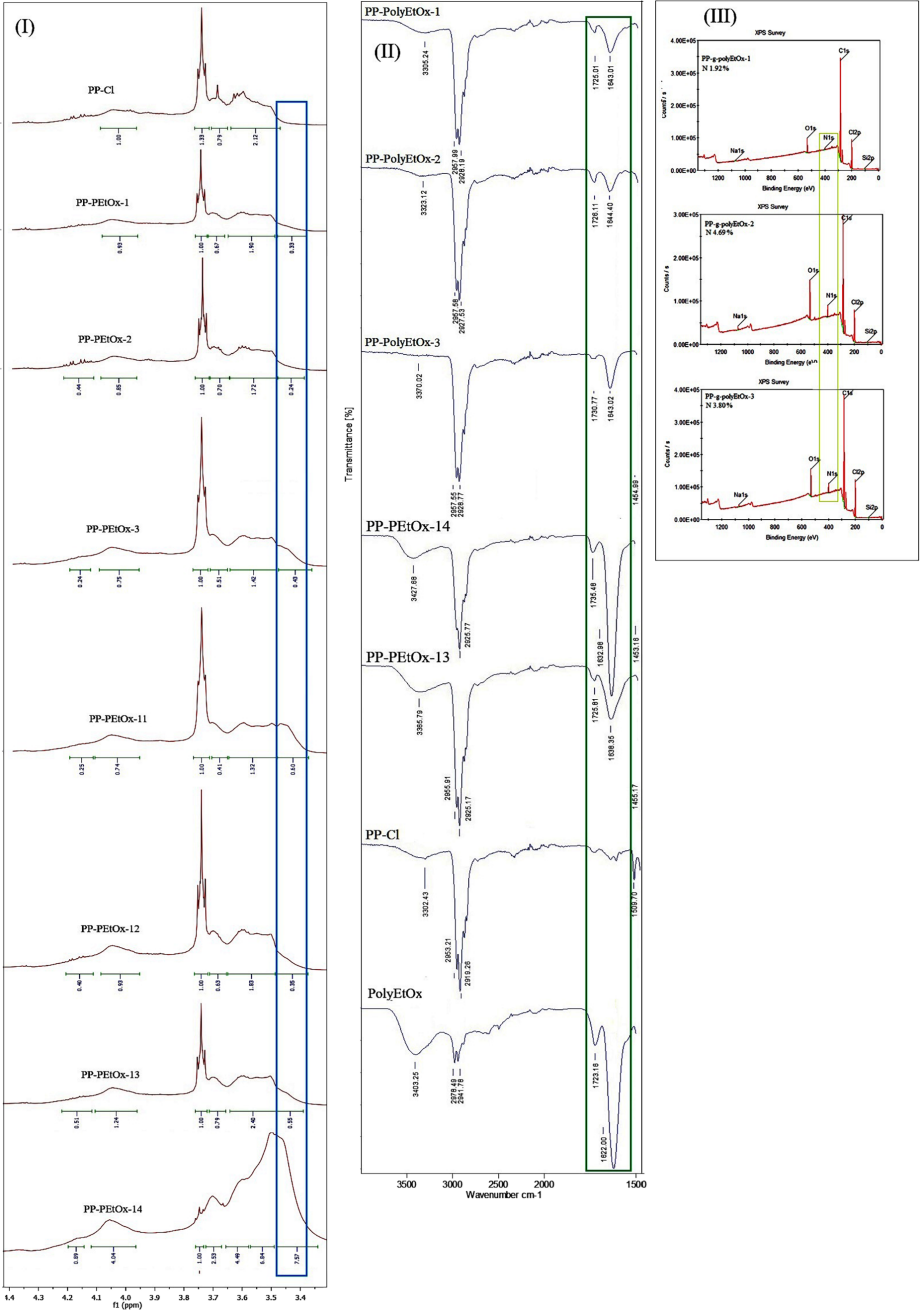
Collective structural characterization of PP–PEtOx graft copolymers: **(I)** 1H NMR spectra showing enlarged characteristic PEtOx signals for PP-Cl, PP-polyEtOx-1, PP-polyEtOx-2, PP-polyEtOx-3, PP-polyEtOx-11, PP-polyEtOx-12, PP-polyEtOx-13, and PP-polyEtOx-14. (II) Characteristic FTIR signals of polyEtOx segments of the graft copolymers, including PP-polyEtOx-1, PP-polyEtOx-2, PP-polyEtOx-3, PP-polyEtOx-14, PP-polyEtOx-13, PP-Cl, and PolyEtOx. (III) XPS spectra containing bond energies of the PP–PEtOx graft copolymers, including PP-PolyEtOx 1, PP-PolyEtOx-2, and PP-PolyEtOx-3.

Figure S2 shows the FTIR spectra of the PP-polyEtOx graft copolymers. The spectrum for PP-Cl displayed the characteristic C–H bands at 2930, 1480, and 1390 cm^–1^, and C–Cl band at 730 cm^–1^. After graft copolymer formations, the presence of polyEtOx in the obtained graft copolymer was also confirmed with the signal of the −N–CH_2_–CH_2_–N– groups at 1643 cm^–1^. Small signal at 1735 cm^–1^ belongs to the −C–O group of the polyEtOx segment. The signals at 2957 and 2928 cm^–1^ are −C–H bands of both the PP and polyEtOx segments. Hydroxyl and hydrophilic groups appeared at 3403 cm^–1^. The C–Cl band was still observed in the spectra of both graft copolymers due to the inactivated C–Cl groups. The characteristic FTIR signals of the polyEtOx segments can be seen in Figure 2II.

Although these results confirmed the graft copolymer formations, the presence of polyEtOx in the block copolymer was also confirmed using XPS analysis. Three polymer samples underwent XPS analysis. Figure 2III shows the XPS analysis results. As part of the elemental analysis, determination of the nitrogen contents was found to be 1.92%, 4.69%, and 3.80% for the PP-*g*-polyEtOx-1, −2, and −3, respectively. Additionally, the chloride band energy at 200 eV indicates the presence of inactivated chloride groups in the block copolymer.

Molar masses (*M*_n_) of the PP-*g*-PolyEtOx copolymers were determined by GPC using linear polystyrene (PS) calibration standards and THF as the eluent. The GPC chromatograms were all unimodal (Figure S3). While widely used, we acknowledge that calibration with linear PS standards does not yield absolute molecular weights for branched or graft copolymers due to differences in hydrodynamic volume between linear PS and PP-*g*-PolyEtOx architectures. Despite these limitations, GPC under these conditions still provides a convenient and reproducible means to compare relative molecular weights within our copolymer series, allowing us to confirm successful grafting by observing shifts in molecular weight distributions relative to those of pristine PP-Cl. The values were between 72 and 112 kDa, being greater than the pristine PP-Cl at 69 kDa (Table 1). For precise molecular weight determination, future studies could employ advanced methods such as multiangle light scattering (MALS) or triple-detection SEC to better account for copolymer architecture.

The water vapor transmission rates (WVTR) were evaluated, and the WVTR of the two membranes were 0.22 and 0.31 g/h·m^2^. These WVTR results are better than those of polyethylene terephthalate membrane (ca. 1.49).^39^

### 3.1 Antibacterial Activity

In recent years, there have been several published articles on the use of poly­(ethyl-2-oxazoline) in the biomedical field. This compound is considered an alternative to polyethylene glycol (PEG), and its potential use as an antibacterial agent has garnered significant interest among researchers.^40–42^ Therefore, the antibacterial responses of PP membranes supplemented with poly (ethyl-2-oxazoline) were tested against both Gram-positive and Gram-negative bacteria. Figures 3 and 4 provide images showing colony counts on the agar. When examining the photographs, it is clearly observed that the membranes functionalized with polyEtOx caused a reduction in colony counts for both *S. aureus* and *E. coli* compared to the control.

**Figure 3:**
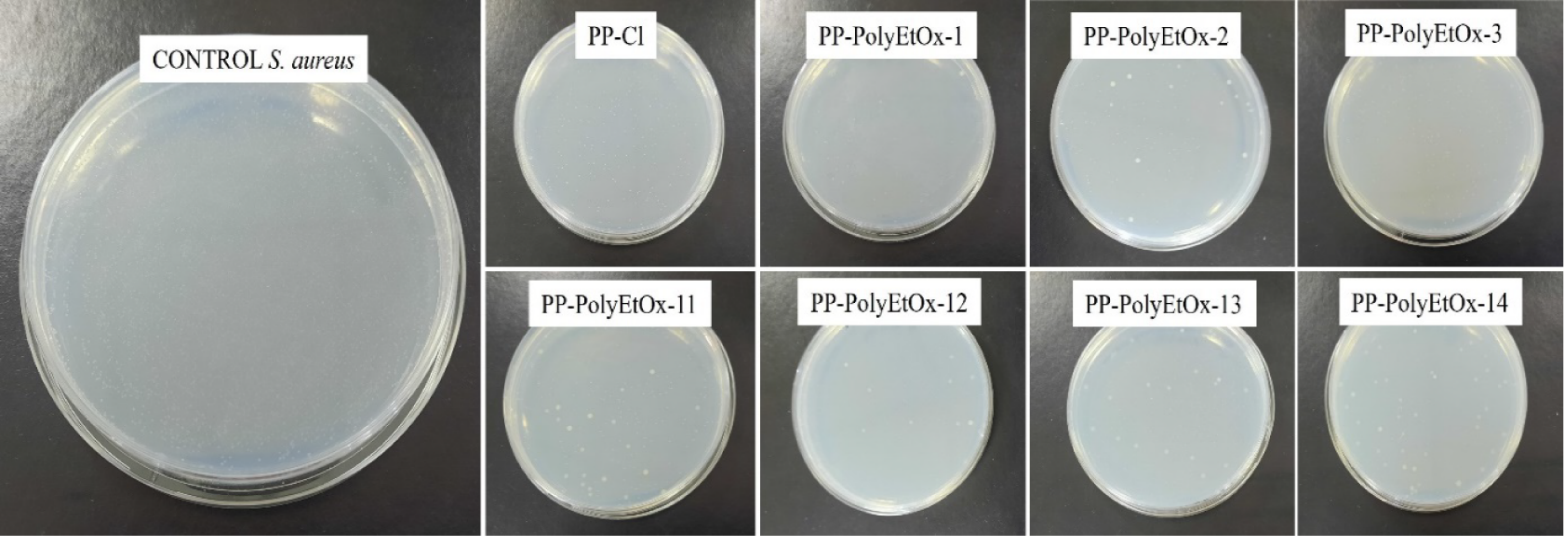
*S. aureus* colony cultures on an agar plate. On agar plates, colonies of *S. aureus* were observed to be significantly reduced when cultured from PP-PolyEtOx membranes compared to the control, as clearly illustrated in the images.

**Figure 4:**
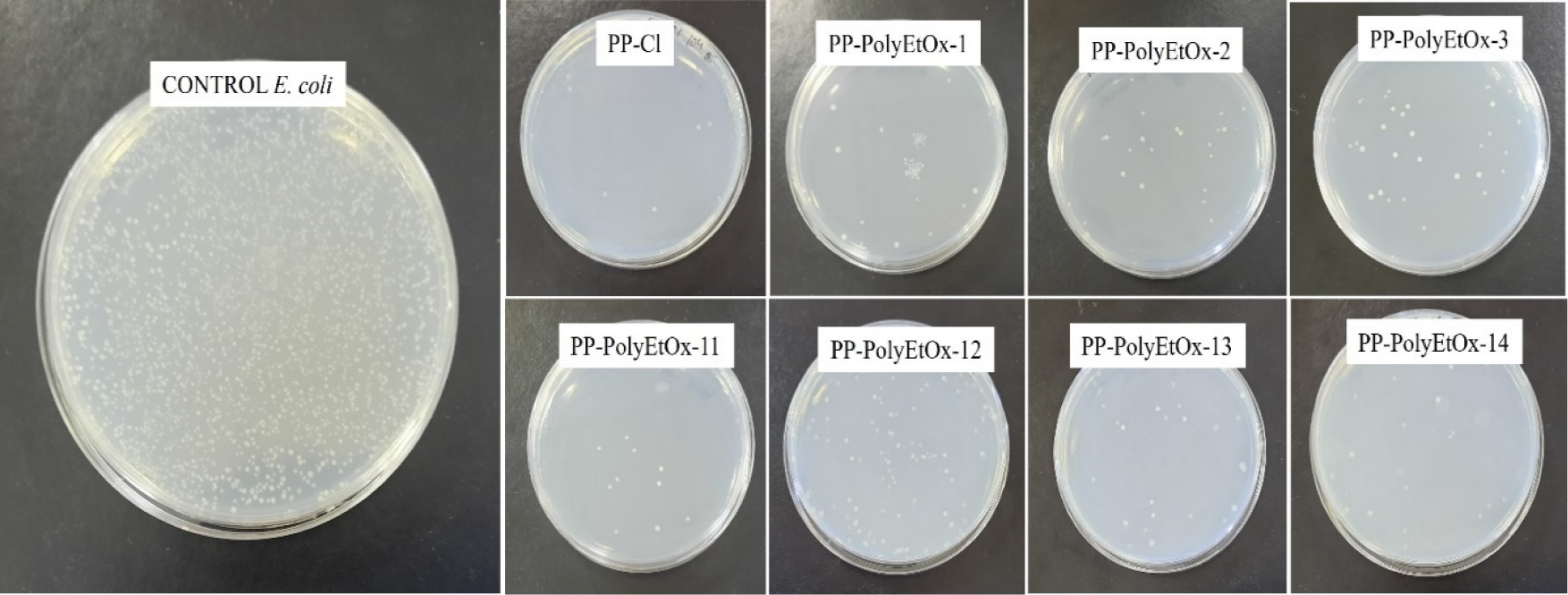
*E. coli* colony cultures on an agar plate. On agar plates, colonies of *E. coli* were observed to be significantly reduced when cultured from PP-PolyEtOx membranes compared to the control, as clearly illustrated in the images.

Numerically comparing colony counts, Figure 5 and Table 2 show log reductions for both bacterial types. For the *S. aureus* bacterial strain, the log reduction in the control at the end of 24 h was 800 CFU × 10^5^ mL^–1^, while for PP-Cl, PP-PolyEtOx-1, PP-PolyEtOx-2, PP-PolyEtOx-3, PP-PolyEtOx-11, PP-PolyEtOx-12, PP-PolyEtOx-13, and PP-PolyEtOx-14, the counts were recorded as 204, 300, 91, 173, 144, 111, 350, and 150 CFU × 10^5^ mL^–1^, respectively. In response to *E. coli* used as the Gram-negative bacterial strain, the log reduction in the control at the end of 24 h was 500 CFU × 10^5^ mL^–1^, while for PP-Cl, PP-PolyEtOx-1, PP-PolyEtOx-2, PP-PolyEtOx-3, PP-PolyEtOx-11, PP-PolyEtOx-12, PP-PolyEtOx-13, and PP-PolyEtOx-14, the counts were 8, 100, 30, 36, 10, 109, 28, and 20 CFU × 10^5^ mL^–1^, respectively. Comparing the responses to both bacterial strains, it is clearly seen that the membranes exhibit better inhibition against *E. coli*. For example, in the group named 14, which has the highest PolyEtOx concentration, the colony count is 150 CFU × 10^5^ mL^–1^, while in the Gram-negative bacterial type, this colony count is observed as 20 CFU × 10^5^ mL^–1^, indicating an approximate 8-fold improvement in colony count. This behavior can be explained by the fact that Gram-positive organisms (such as *S. aureus*) have a thicker cell wall. Additionally, the long hydrophobic chain of PP-PolyEtOx might have improved bacterial adhesion. We know that superhydrophilic (WCA 0°) or superhydrophobic surfaces (WCA 168°) prevent bacterial adhesion.^43^

**Figure 5:**
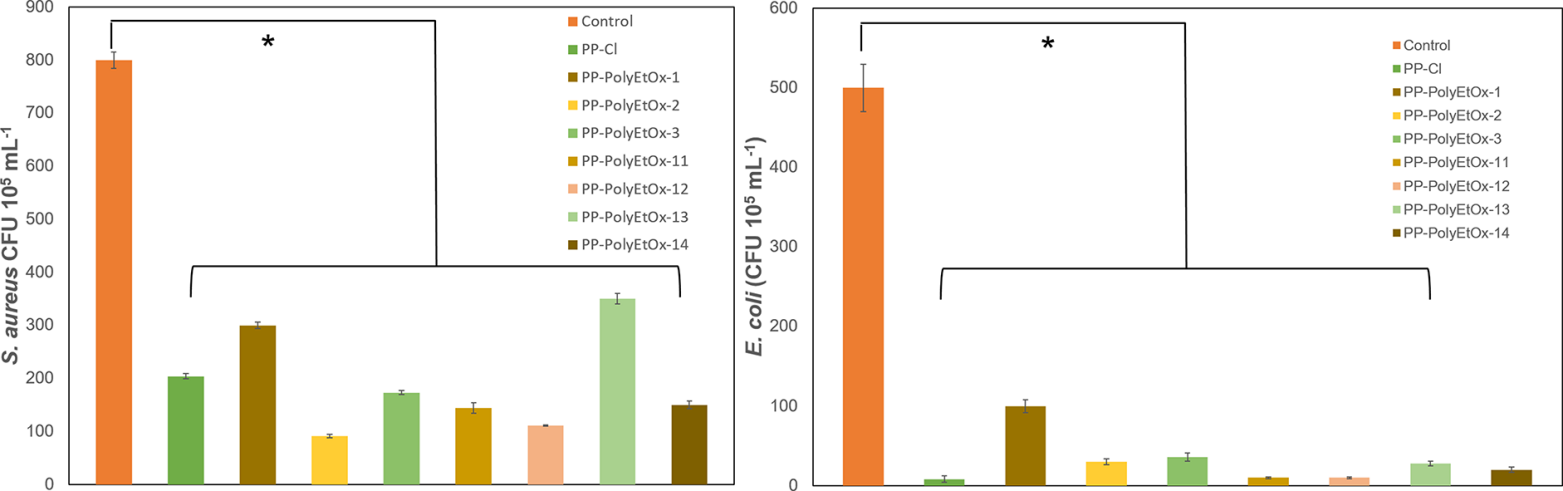
Antimicrobial activity (number of colony-forming units per milliliter, CFU/mL) of PP-PolyEtOx membranes on the *S. aureus* and *E. coli* strains. Values are presented as mean ± SEM; *n* = 3. PP-membranes showed a significant difference compared to control (*p < 0.005).

**Table 2:** *S. aureus* and *E. Coli* Counts (Number of Colony-Forming Units per Milliliter, CFU/mL) on the PP-PolyEtOx Membranes

*S. aureus*		****E. coli****	
Samples	CFU (10^5^ mL^–1^)	Samples	CFU (10^5^ mL^–1^)
Control	800	Control	500
PP-Cl	204	PP-Cl	8
PP-PolyEtOx-1	300	PP-PolyEtOx-1	100
PP-PolyEtOx-2	91	PP-PolyEtOx-2	30
PP-PolyEtOx-3	173	PP-PolyEtOx-3	36
PP-PolyEtOx-11	144	PP-PolyEtOx-11	10
PP-PolyEtOx-12	111	PP-PolyEtOx-12	10
PP-PolyEtOx-13	350	PP-PolyEtOx-13	28
PP-PolyEtOx-14	150	PP-PolyEtOx-14	20

Discussing the effect of PolyEtOx concentration on the antibacterial response in both bacterial types, a significant improvement has been noted. For example, on PP-PolyEtOx-14 membranes (feeding concentration 60% PolyEtOx), only 150 CFU × 10^5^ mL^–1^ *S. aureus* colonies were observed, whereas the colony counts on sample number 13, which has 21% PolyEtOx content, are 350 CFU × 10^5^ mL^–1^. However, in the case of *E. coli*, no significant difference was found between the PolyEtOx concentration and the improvement in colony count. Chlorinated polypropylene also shows antibacterial activity. These obtained membranes still contain unreacted chloride pendant groups and show a similar antibacterial effect. Nonetheless, they still maintain an antibacterial effect for active food packaging applications.

### 3.2 Flow Cytometry Analysis

The effects of the PP-PolyEtOx membranes on the activation of HT-29 cell apoptosis are presented in Figure 6 and Table 3. Recent studies show that synthetic Cl ion carriers disrupt cellular ion homeostasis and induce apoptosis in cancer cells.^44^ Based on this information, as expected, the group with the highest apoptosis rate was PP-Cl. Interestingly, instead of the highest PolyEtOx concentration of 14, the lowest PolyEtOx concentrations of 11 and 13 showed the highest percentages of apoptosis, with early apoptosis rates of 5.12% and 9%, respectively. As detected in the ROS analysis results, PolyEtOx induces apoptosis in cancer cells regardless of concentration. This means that lower PolyEtOx concentrations are more effective in cancer cells.

**Figure 6:**
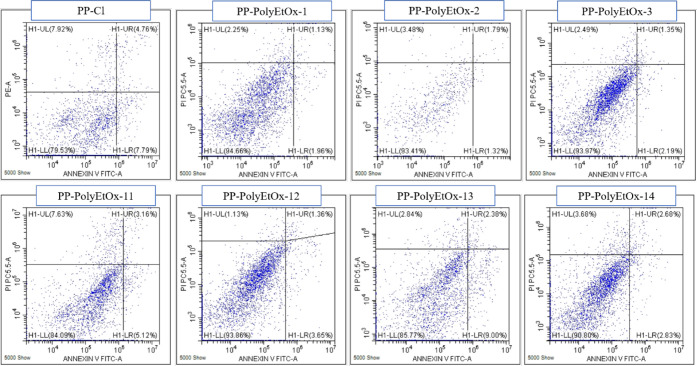
Flow cytometry analysis of HT-29 cells exposed to PP-PolyEtOx membranes. H1-UL, H1-UR, H1-LL, and H1-LR dot plots showed necrotic cells (N, Annexin V–/PI+), late- and secondary-apoptotic cells (LA, Annexin V+/PI+), living cells (LC, Annexin V–/PI−), and early- and primary-apoptotic cells (EA, Annexin V+/PI−), respectively.

**Table 3:** Necrotic, Late/Secondary Apoptotic, Living, and Early/Primary Apoptotic Cell Percentages in HT-29 Cell Cancer Cells Incubated with PP-PolyEtOx Membranes

Samples	LL (%)	UL (%)	LR (%)	UR (%)
PP-Cl	79.53	7.92	5.66	4.76
PP-PolyEtOx-1	94.66	2.25	1.96	1.13
PP-PolyEtOx-2	93.41	3.48	1.32	1.79
PP-PolyEtOx-3	93.97	2.49	2.19	1.35
PP-PolyEtOx-11	84.09	7.63	5.12	3.16
PP-PolyEtOx-12	93.86	1.13	3.65	1.36
PP-PolyEtOx-13	85.77	2.84	9	2.38
PP-PolyEtOx-14	90.8	3.68	2.83	2.68

He and colleagues previously developed amphiphilic poly­(2-oxazoline) block copolymers formulated into micelles for delivering a third-generation taxoid (PolyEtOx/SB-T-1214), reporting improved anticancer efficacy against multidrug-resistant (MDR) cells compared to free paclitaxel.^45^ While micelle-based formulations represent a distinctly different system—a solution-based nanoscale carrier as opposed to our bulk membrane system—we reference them here to underscore the general biomedical potential of polyEtOx. Our PP-PolyEtOx graft copolymer membranes differ significantly in physical state and application focus; nevertheless, the observed cytotoxicity and ROS generation results indicate that polyEtOx segments retain anticancer functionality even outside a micellar context. We explicitly avoid making a direct comparison between membrane-based and micellar systems, given their fundamentally different mechanisms of cellular interaction and therapeutic delivery. Instead, the prior micellar research broadly supports the notion that polyEtOx-containing materials hold promise for advanced biomedical applications, whether in solution-based carriers or solid-phase membranes.

PolyEtOx are attractive for biomedical applications due to similar characteristics to the “gold standard” PEG, and have many superior features such as weak interactions with human serum proteins, antimicrobial effect, chemical versatility, nontoxicity, high stability, and low immunogenicity to overcome PEG’s limitations.^46–48^

Poly (2-ethyl-2-oxazoline) (PEtOx) is an exciting platform for the construction of amphiphilic block copolymer nanoparticles and, subsequently, for delivering antitumor drugs in biomedical applications. Thus, the ROS production induced with the PP membranes containing PolyEtOx at different concentrations was evaluated. For this experiment, the DCF-DA fluorescence probe was used, and ROS production in treated HT-29 cells was measured as 2.21 , 3.43, 2.07, 4.24, 25.06, 2.42, 21.79, and 48.08% for PP-Cl, PP-PolyEtOx-1, -2, -3, -11, -12, -13, and -14, respectively (Figure 7 and Table 4). The results showed that ROS production significantly increased at PP-PolyEtOx-11, PP-PolyEtOx-13, and PP-PolyEtOx-14. Among the samples, PP-PolyEtOx-14 has the highest PolyEtOx concentration, and the order related to PolyEtOx concentration is as follows: 14 > 2 > 1 = 3 = 12 > 11 = 13. PP-PolyEtOx-14 induced ROS formation in a concentration-dependent manner. However, it is interesting to note that PP-PolyEtOx-11 and PP-PolyEtOx-13, at low concentrations (21% and 22%, respectively), also induced higher ROS levels compared to the other PP-PolyEtOx groups (PP-Cl, and PP-PolyEtOx-1, -2, -3, and -12). H2DCFDA fluorescence signals of PP-Cl, PP-PolyEtOx-1, -2, -3, and -12 were found to be very close to each other.

**Figure 7:**
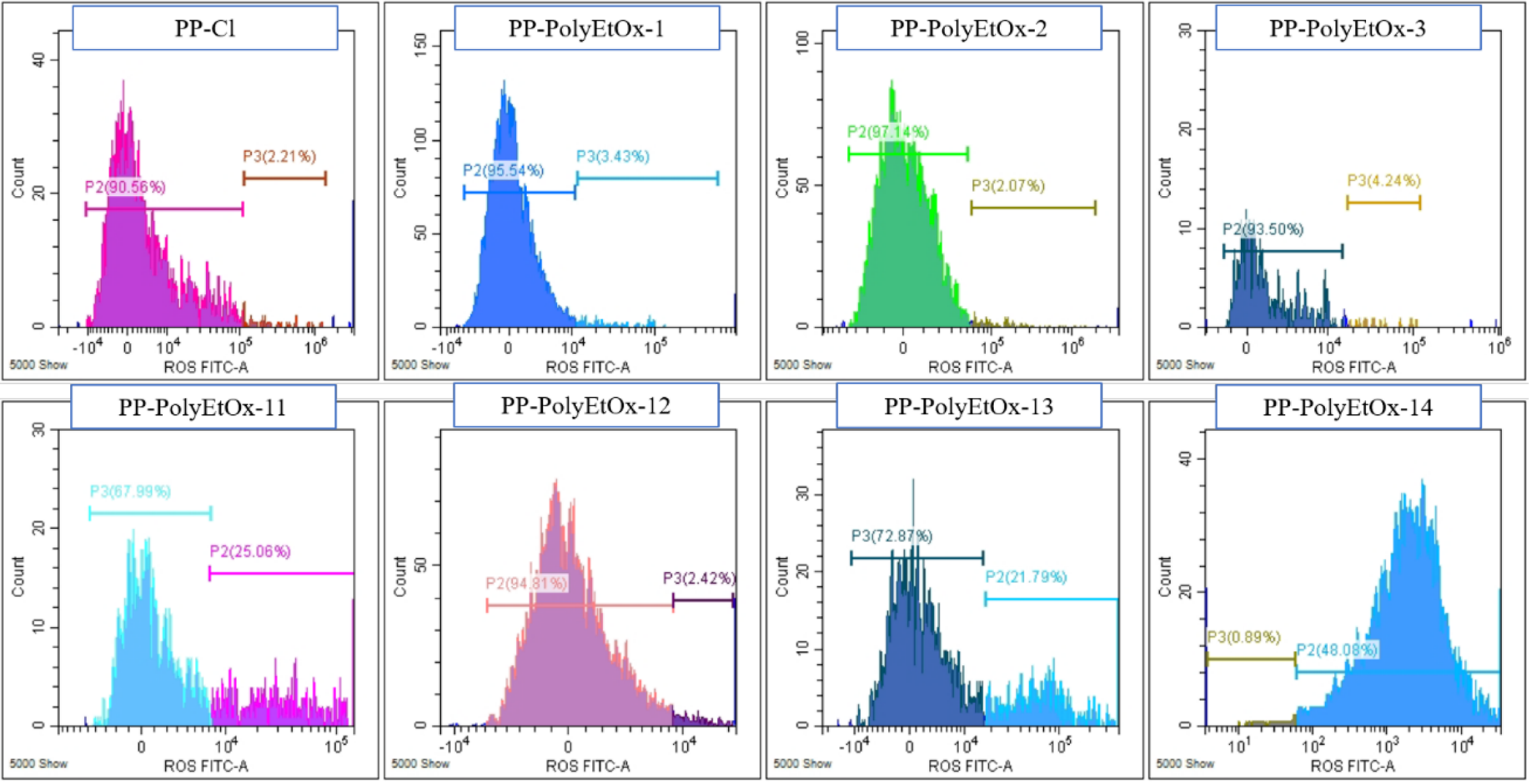
Flow cytometry histograms of ROS production in HT-29 cells and cancer cells incubated with PP-PolyEtOx membranes.

**Table 4:** H2DCFDA Fluorescence Signal Intensity (%) in HT-29 Cancer Cells Incubated with PP-PolyEtOx Membranes

Samples	ROS FITC (%)
PP-Cl	2.21
PP-PolyEtOx-1	3.43
PP-PolyEtOx-2	2.07
PP-PolyEtOx-3	4.24
PP-PolyEtOx-11	25.1
PP-PolyEtOx-12	2.42
PP-PolyEtOx-13	21.8
PP-PolyEtOx-14	48.1

Knop et al. reported poly­(2-oxazoline) block copolymers with diverse architectures and molar masses that exhibited reduced cell viability at elevated concentrations, suggesting that increased polymer hydrophobicity may accentuate toxicity.^46^ Similarly, in our study, we observed enhanced apoptotic activity in HT-29 cells at higher polyEtOx content (e.g., PP-PolyEtOx-14). However, it is important to emphasize that cytotoxic effects at very high polymer concentrations often reflect nonspecific cellular stress rather than intrinsic polymer toxicity-a phenomenon common to virtually all polymers, including those generally regarded as biologically inert. Therefore, the notable apoptosis and ROS generation that we observed at moderate polyEtOx feed ratios more accurately reflect the specific biological interactions and underscore the potential biomedical relevance of these graft copolymers, distinct from general cytotoxicity arising solely from excessive polymer dosage.

## 4 Conclusions

Polypropylene is a plastic with excellent and desirable properties, such as good elasticity and mechanical strength. Its major disadvantage is its non-biodegradability . However, vinyl plastics have still been used as food packaging material. The modification reactions of the vinyl plastics using hydrophilic and biologically active compounds make them membraneswidely used in food packaging applications. Water vapor transmission rates of the two membranes were 0.22 and 0.31 g/h·m^2^. These WVTR results are better than those of the polyethylene terephthalate membrane. PolyEtOx-functionalized polypropylene was found to have good anticancer and antibacterial biological properties. In addition to this, graft copolymer of hydrophobic polypropylene and hydrophilic PolyEtOx is very attractive in solution and micelle properties for physical chemists. Additionally, flow cytometry and ROS detection experiments revealed that the synthesized PP-PolyEtOx membranes could induce apoptosis in HT-29 colon cancer cells, as indicated by elevated ROS levels. These preliminary results suggest that PP-PolyEtOx membranes may possess both antibacterial and anticancer potential.

Further comprehensive studies—including detailed toxicity assessments, mechanism-of-action evaluations, and in vivo experiments—would help substantiate their suitability for advanced biomedical and food packaging applications. Furthermore, due to the similarity of the oxazoline content to the structure of PEG, these membranes have the potential to expand their application areas, clearly demonstrating their versatility for diverse uses. For future work, these amphiphilic copolymers can also be used in releasing the bioactive adducts to the surface of food. Preparation of some nanocomposite membranes will be attractive for the nanotechnological applications, including quantum dot systems and related polymerization kinetics.^49–52^ The cost-effective vinyl polymers can be functionalized with several different, more natural bioactive compounds for new food packaging applications. We recognize that achieving an optimal balance of structural integrity, mechanical performance, and biological compatibility remains a significant challenge in polymer science, and this study provides a preliminary step toward addressing that need.

## Supplementary Material



## Data Availability

The original contributions presented in the study are included in the article; further inquiries can be directed to the corresponding author.

## Notes

^1^H NMR spectra (SI-Figure 1), FTIR spectra (SI-Figure 2), and GPC chromatograms (SI-Figure 3) of the polyEtOx graft copolymers (PDF)
