# Supplementary material for: Synthesis and Characterization of a Novel Membrane Based on Poly (2-Ethyl Oxazoline) and Poly (Propylene) Graft Copolymer for Potential Food Packaging and Medical Applications
Source: ACS Omega. 2025 Aug 4;10(31):34227–37. doi: 10.1021/acsomega.5c00605 (PMC12355271; doi:10.1021/acsomega.5c00605)
Supplement: Supplementary file 1 [file ao5c00605_si_001.pdf]

SUPPORTING INFORMATION

Synthesis and characterization of a novel membrane based on Poly (2-Ethyl Oxazoline) and Poly (propylene) graft copolymer for potential food packaging and medical applications

Baki Hazer [1,2], Zeynep Karahaliloglu [3], Özgür Keleş [4,*]

[1]Kapadokya University, Department of Aircraft Airframe Engine Maintenance, Mustafapaşa Kasabası 50420 Ürgüp, Nevşehir Turkey.

[2]Zonguldak Bülent Ecevit University, Departments of Chemistry/Nano Technology Engineering, 67100 Zonguldak, Turkey.

[3]Department of Biology, Faculty of Science, Aksaray University, 68000 Aksaray, Turkey

[4]Department of Mechanical Engineering and Engineering Science, University of North Carolina at Charlotte, Charlotte, NC 28223

*Ozgur Keles, okeles@charlotte.edu

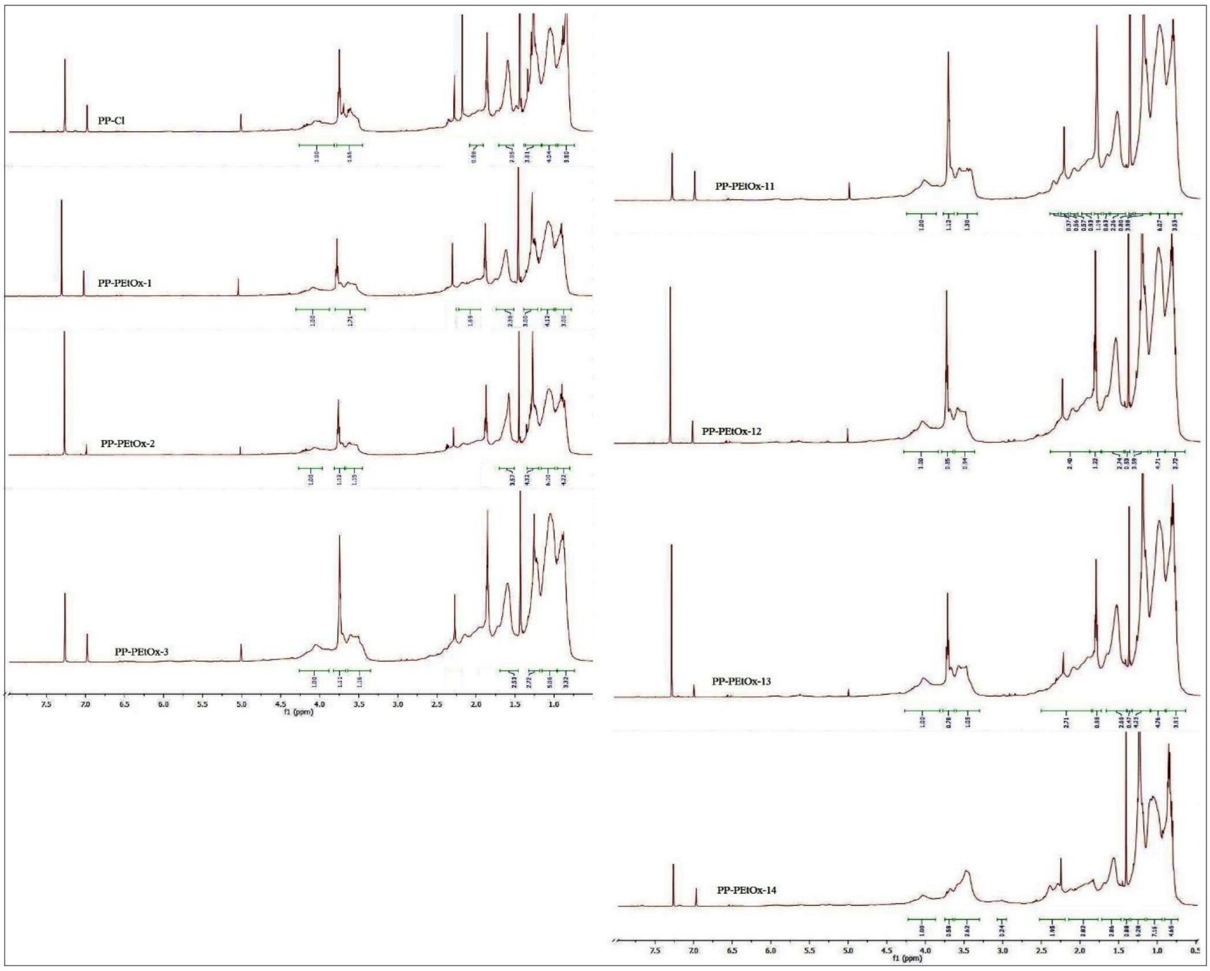

SI-Figure 1 ^{1}H NMR spectra of the as synthesized PP-g-PolyEtOx graft copolymers.

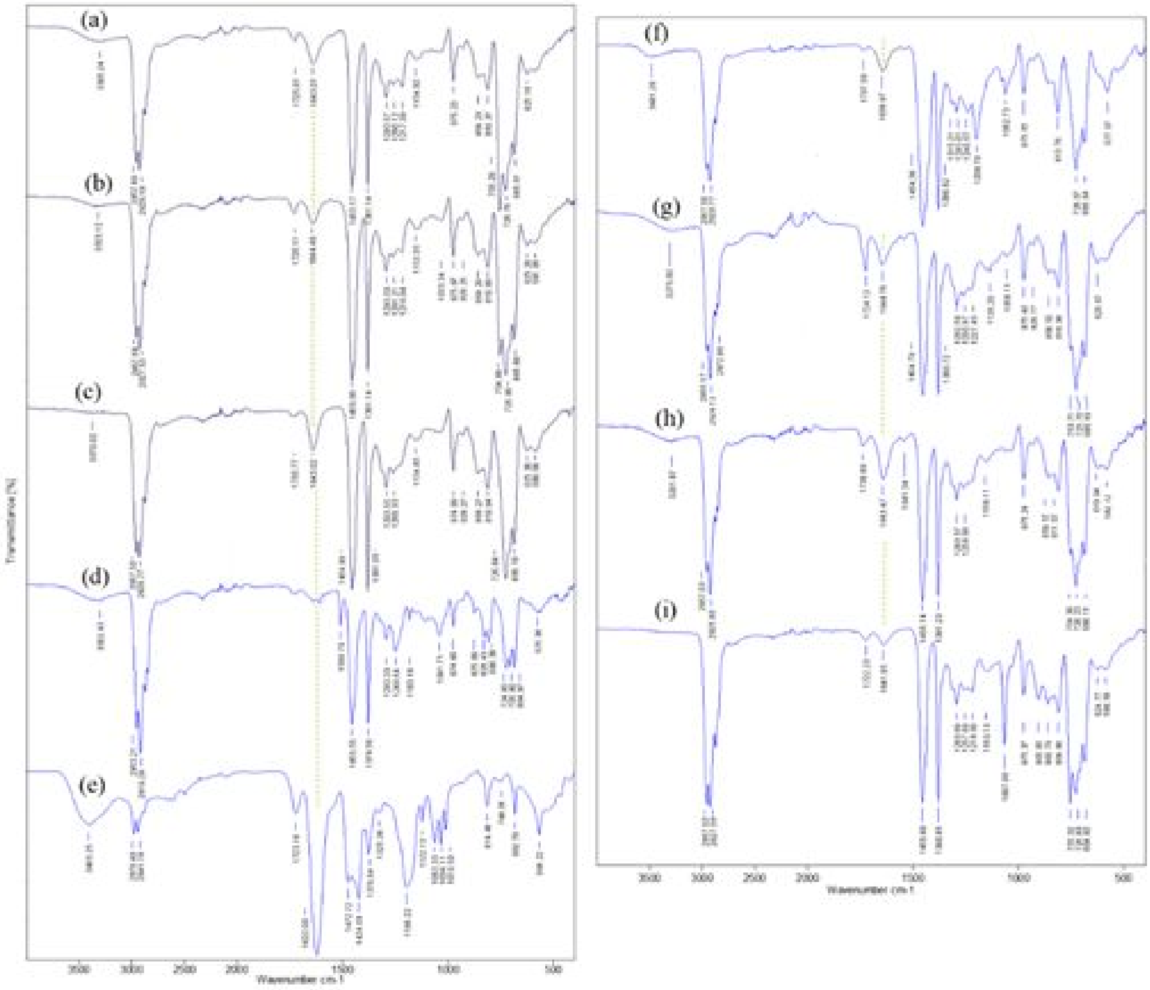

SI-Figure 2. FTIR spectra of the PP-polyEtOx graft copolymers: (a) PP-polyEtOx-1, (b) PP-polyEtOx-2, (c) PP-polyEtOx-3, (d) PP-Cl, (e) PolyEtOx, (f) PP-polyEtOx-11, (g) PP-polyEtOx-12, (h) PP-polyEtOx-13, (i) PP-polyEtOx-14.

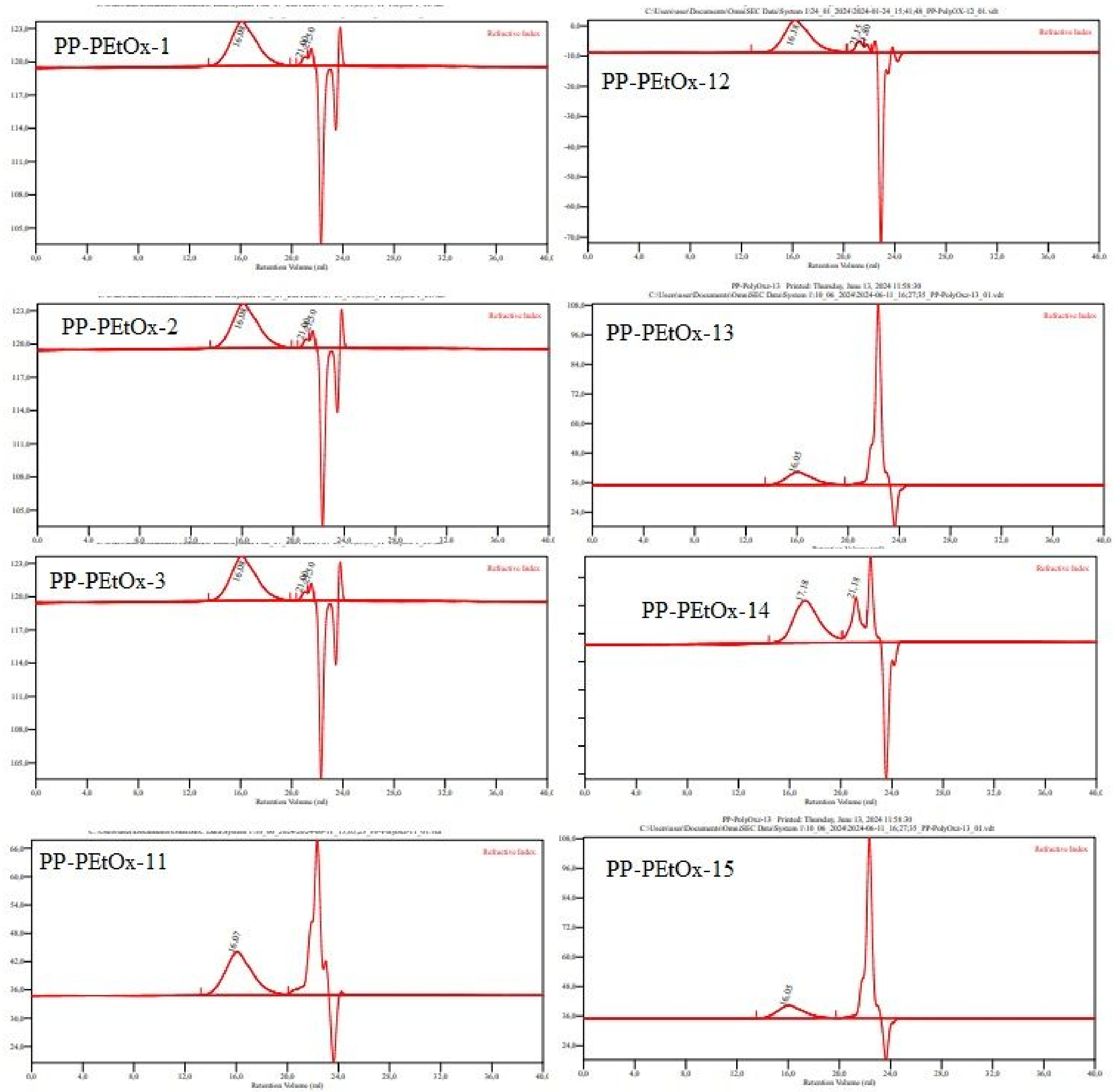

SI-Figure 3. GPC chromatograms of the PP-PEtOx derivatives.
